# Myocardial extracellular volume derived from contrast-enhanced chest computed tomography for longitudinal evaluation of cardiotoxicity in patients with breast cancer treated with anthracyclines

**DOI:** 10.1186/s13244-022-01224-5

**Published:** 2022-05-04

**Authors:** Chunrong Tu, Hesong Shen, Renwei Liu, Xing Wang, Xiaoqin Li, Xiaoqian Yuan, Qiuzhi Chen, Yu Wang, Zijuan Ran, Xiaosong Lan, Xiaoyue Zhang, Meng Lin, Jiuquan Zhang

**Affiliations:** 1 Department of Radiology, Chongqing University Cancer Hospital, 181 Hanyu Road, Chonqing, 400030 China; 2 Siemens Healthineers, Xi’an, China

**Keywords:** Myocardium, Extracellular volume, Anthracyclines, Cardiotoxicity, Computed tomography

## Abstract

**Objectives:**

To assess the value of myocardial extracellular volume (ECV) derived from contrast-enhanced chest computed tomography (CT) for longitudinal evaluation of cardiotoxicity in patients with breast cancer (BC) treated with anthracycline (AC).

**Materials and methods:**

A total of 1151 patients with BC treated with anthracyclines, who underwent at least baseline, and first follow-up contrast-enhanced chest CT were evaluated. ECV and left ventricular ejection fraction (LVEF) were measured before (ECV_0_, LVEF_0_), during ((ECV_1_, LVEF_1_) and (ECV_2_, LVEF_2_)), and after (ECV_3_, LVEF_3_) AC treatment. ECV values were evaluated at the middle of left ventricular septum on venous phase images. Cancer therapy-related cardiac dysfunction (CTRCD) was recorded.

**Results:**

Mean baseline LVEF values were 65.85% ± 2.72% and 102 patients developed CTRCD. The mean ECV_0_ was 26.76% ± 3.03% (N_0_ = 1151). ECV_1_, ECV_2_, and ECV_3_ (median interval: 61 (IQR, 46–75), 180 (IQR, 170–190), 350 (IQR, 341–360) days from baseline) were 31.32% ± 3.10%, 29.60% ± 3.24%, and 32.05% ± 3.58% (N_1_ = 1151, N_2_ = 841, N_3_ = 511). ECV_1_, ECV_2_, and ECV_3_ were significantly higher than ECV_0_ (*p* < 0.001). ECV_0_ and ECV_1_ showed no difference between CTRCD (+) and CTRCD (−) group (*p*_1_ = 0.150; *p*_2_ = 0.216). However, ECV_2_ and ECV_3_ showed significant differences between the two groups (*p*_3_ < 0.001; *p*_4_ < 0.001).

**Conclusion:**

CT-derived ECV is a potential biomarker for dynamic monitoring AC cardiotoxicity in patients with BC.

## Key points

- CT-derived extracellular volume (ECV) increased after anthracycline treatment in breast cancer (BC) patients.
- ECV showed difference between BC patients with and without cancer therapy-related cardiac dysfunction (CTRCD).
- CT-derived ECV is a potential biomarker for monitoring anthracycline cardiotoxicity in BC

## Introduction

Anthracyclines (ACs) are highly effective chemotherapeutic agents frequently used in the treatment of breast cancer (BC) [1]. However, cardiotoxicity is a prominent undesirable effect of anthracycline therapy that causes diffuse and irreversible myocardial fibrosis [2–4]; the frequency and severity of cardiotoxicity depend on the accumulated dose of anthracycline. Cardiotoxicity is known to be associated with adverse cardiac events and severely affects the prognosis of cancer survivors [5, 6].

Recently, extracellular volume (ECV), measured by cardiac magnetic resonance (CMR) T1-mapping, has been developed to quantify the diffuse myocardial fibrosis caused by chemotherapy-induced cardiotoxicity [7–9]. The ECV fraction reflects the percentage of myocardium not constituted by cells and increases when there is deposition of a pathological substance, as in fibrosis or amyloidosis [10, 11]. Several studies have evaluated ECV as a biomarker of anthracycline-induced cardiotoxicity using CMR T1-mapping before and after the administration of gadolinium-based contrast agent [8, 12]. However, CMR has some limitations and contraindications that limit its clinical application; for example, it is expensive, time-consuming, and can induce claustrophobia. Computed tomography (CT) has some disadvantages of radiation risks and CT contrast agent damage. However, it also has the advantages of high spatial resolution and wide application, and has been gradually used to characterize myocardium structure. Recent studies have proposed CT-derived ECV for the detection of diffuse myocardial fibrosis [13, 14]. The rationale for these studies is that iodine contrast agents used in CT and gadolinium contrast agents used in CMR have similar dynamics, molecular weight, and ECV distributions despite different molecular structures [15]. In addition, several studies have shown that CT-derived ECV is strongly correlated with CMR-derived ECV and histology [14].

In recent years, with the prolonged survival time of patients with BC, cancer therapy-related cardiac dysfunction (CTRCD) has become the most important type of cardiotoxicity, as it severely impacts prognosis and quality of life in BC survivors [16]. Currently, imaging methods are far from optimal for identifying anthracycline-induced cardiotoxicity; left ventricular ejection fraction (LVEF) is most commonly used to monitor cardiac function in patients treated with anthracyclines [17]. However, by the clinical stage at which LVEF declines, damage to the heart is often irreversible. Therefore, to mitigate the risk of CTRCD, early detection and monitoring of cardiotoxicity caused by anthracyclines is essential.

Therefore, the objective of this study was to assess myocardial ECV derived from contrast-enhanced chest CT for longitudinal evaluation of cardiotoxicity in patients with BC treated with anthracycline.

## Materials and methods

### Study population

**Fig. 1:**
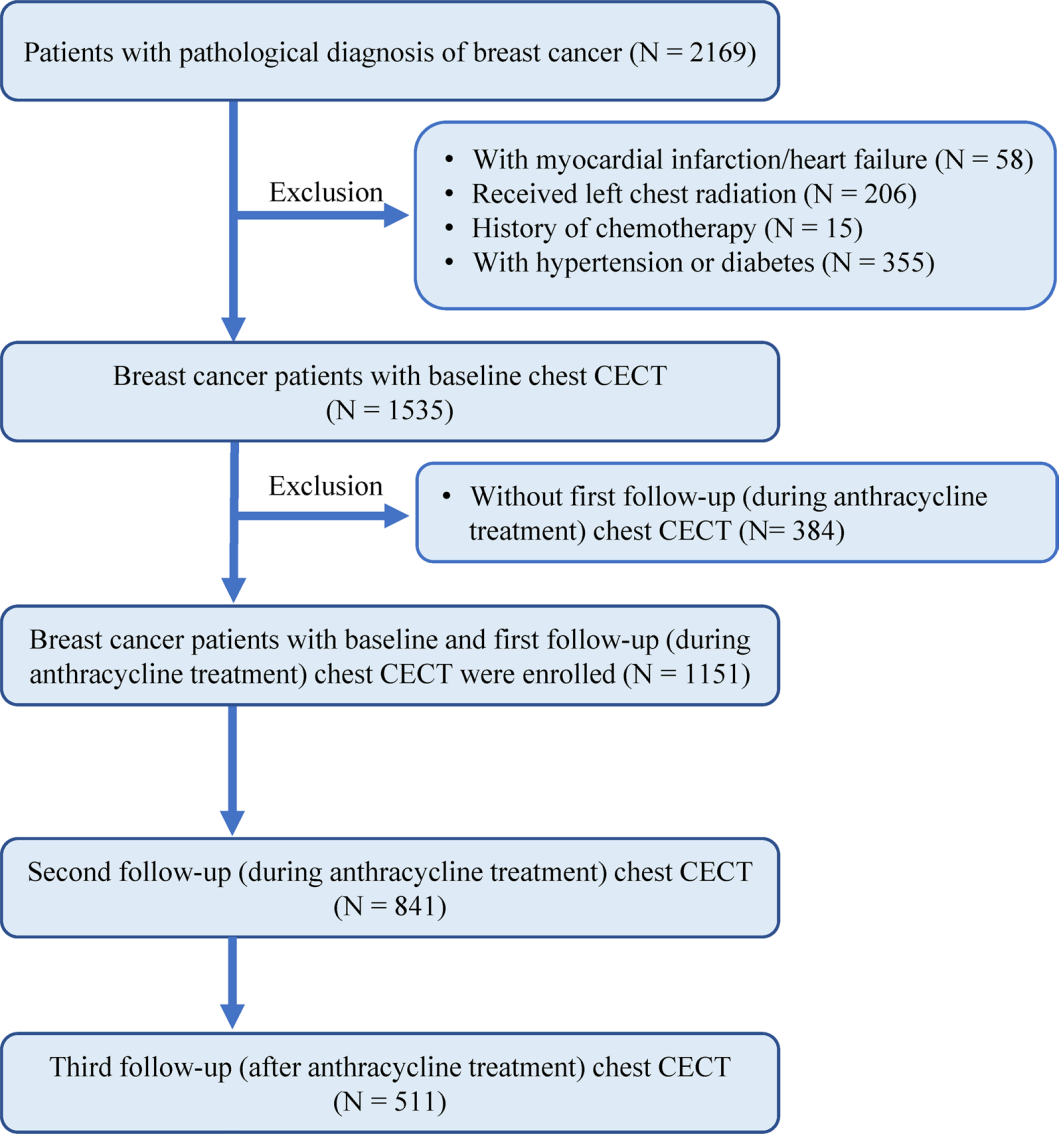
Flow diagram of participants. *CECT*, contrast-enhanced computed tomography

This study was approved by our institution's ethics committee. The need for written informed consent was waived because of the retrospective study design. From December 2014 to March 2021, 2169 patients with a pathological diagnosis of BC underwent contrast-enhanced chest CT for the evaluation of potential lung metastases or concomitant pulmonary disease. Two-dimensional echocardiography (2DE) was performed to monitor cardiac function both before and after treatment. These patients were retrospectively selected through a CT database and each patient’s medical history and relevant clinical characteristics were recorded by a radiologist with 15 years of experience. The inclusion criteria were as follows: (1) pathologically confirmed diagnosis of BC; (2) treated with AC chemotherapy according to guidelines [18]; (3) underwent at least two chest contrast-enhanced CT examinations, including baseline (before AC treatment) and first follow-up (during AC treatment). Exclusion criteria were as follows: (1) received radiotherapy for left BC due to its potential for confounding cardiotoxicity; (2) previously received chemotherapy; (3) history of active or prior cardiac disease (e.g., myocardial infarction, heart failure, primary myocardiopathy, valvular heart disease or cardiac sarcoidosis); (4) had cardiac risk factors, such as hypertension or diabetes. Fifty-eight elderly patients had a history of myocardial infarction or heart failure. Two hundred and six patients received left chest wall radiation for left BC. Fifteen patients had a prior history of chemotherapy. Three hundred and fifty-five patients had hypertension or diabetes. Three hundred and eighty-four patients had only baseline and no follow-up chest contrast-enhanced CT examinations. Finally, 1151 patients with BC treated with AC chemotherapy were included. The study flowchart is shown in Fig. 1.

### CT acquisition protocols

Contrast-enhanced chest CT is recommended for patients with recurrence or suspected lung metastasis according to the guidelines for the clinical management of BC [18]. CT examinations were performed on two CT scanners (SOMATOM Definition AS + , Siemens Healthineers, Germany; SOMATOM Drive, Siemens Healthineers, Germany). Patients were instructed to hold their breath during acquisition, and all scans were performed in a craniocaudal (top-down) direction. SOMATOM Definition AS + and SOMATOM Drive parameters were as follows: tube voltage, 120 kVp; reference tube current, 110 mAs; detector collimation, 0.625–1.5 mm; beam pitch, 1.375–1.5. Image data were reconstructed with 1.0-mm thick sections with the mediastinum algorithm. All patients underwent total chest CT scans that included the heart.

An iodinated contrast agent (Ioversol, 320 mg/mL iodine, HENGRUI Medicine, Jiangsu, China) was injected intravenously at a dose of 1.5 mL/kg and a flow rate of 2.5 mL/sec, followed by 30 mL of saline solution at the same flow rate.

Enhanced scanning is determined using automatic exposure control (CARE Dose 4D, Siemens Healthineers, Forchheim, Germany). To be specific, low-dose monitoring images were obtained on uniaxial sections of the aorta after contrast agent injection. After injection, arterial phase scans were started using a bolus-tracking technique with a threshold of 100 Hounsfield units (HU) in the descending aorta. The portal phase scan was performed approximately 80 s after the descending aorta reached 100 HU.

### CT Image analysis

**Fig. 2:**
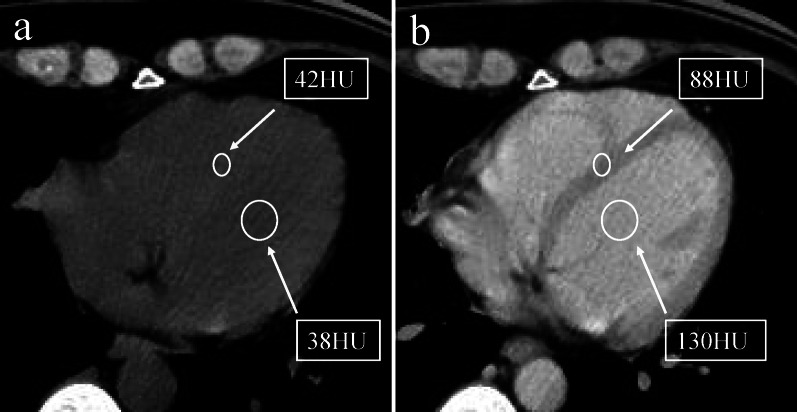
Region of interest for extracellular volume calculations on computed tomography in a 55-year-old woman with breast cancer. **a** Contrast-enhanced scan, the ROIs were manually delineated in the middle of the interventricular septum and blood pool avoiding the papillary muscle. **b** Unenhanced scan, copy the two ROIs in the contrast-enhanced image and place them at similar locations in the unenhanced ventricular septum and blood pool. *ROI*, region of interest

All images were independently evaluated by two radiologists with 10 and 15 years of experience. First, the observer selected the slice that allowed the best longitudinal visualization of left ventricular septum on the portal phase images. Second, the region of interest (ROI) was manually delineated in the middle of the interventricular septum. Next, another ROI was drawn in the cardiac blood pool of the left ventricle at the same level, avoiding the papillary muscle as much as possible. Then, the two ROIs drawn on the portal phase image in the previous steps were duplicated and placed at similar locations in the unenhanced ventricular septum and blood pool (Fig. 2) [19]. Due to the effects of free breathing cardiac activity, the ROI was placed in the middle of the ventricular septum.

According to the previous study [20], septal ECV was calculated using the following formula:

$$\mathrm{ECV}=\left(1-\mathrm{HCT}\right)\times \left[\left({\mathrm{HU}}_{{\mathrm{myo}}_{\mathrm{post}}}-{\mathrm{HU}}_{{\mathrm{myo}}_{\mathrm{pre}}}\right)/\left({\mathrm{HU}}_{{\mathrm{blood}}_{\mathrm{post}}}-{\mathrm{HU}}_{{\mathrm{blood}}_{\mathrm{pre}}}\right)\right]$$

where myo = myocardium; pre = pre-contrast; and post = post-contrast.

### LVEF acquisition and CTRCD definition

All patients had LVEF measured by 2DE (Philips Ultrasound System, Q7, Philips Healthcare) within 7 days before or after CT scan.

CTRCD was defined as a ≥ 10% reduction in LVEF from baseline to < 55% by 2DE during the follow-up, according to the American Society of Echocardiography and the European Association of Cardiovascular Imaging expert consensus [21].

### Statistical analysis

SPSS ver. 25.0 (IBM Institute, Armonk, NY, USA) was used for all statistical analyses. The Kolmogorov–Smirnov test was used to assess the normality of the quantitative data distribution. Continuous data were summarized as the mean ± SD or median and interquartile range (IQR) as appropriate; categorical data were expressed as frequencies or percentages. Comparisons between patients with and without CTRCD were performed using Student’s t tests or Chi-square tests. The differences among ECVs at baseline and different follow-up times were compared using a one-way analysis of variance with the Bonferroni post hoc test. The longitudinal variation of ECV with time was linearly fitted. The intra- and interobserver reproducibility of CT-derived ECVs were assessed by the interclass correlation coefficient (ICC). ICC was calculated to evaluate interobserver agreement for the ECV fraction (ICC = 0.00–0.20, poor correlation; ICC = 0.21–0.40, fair correlation; ICC = 0.41–0.60, moderate correlation; ICC = 0.61– 0.80, good correlation; ICC = 0.81–1.00, excellent correlation). Bland–Altman analyses were conducted to evaluate data consistency. In all other tests, a two-sided *p* value of < 0.05 was considered to indicate a significant difference.

## Results

### Study population

After the application of exclusion criteria, a total of 1151 patients with BC were included in the study cohort; chemotherapy dose was adjusted for body weight and body surface area according to clinical guidelines. Baseline patient characteristics are summarized in Table 1. Patients underwent chest contrast-enhanced CT examinations before anthracycline treatment ("baseline"), during and after anthracycline therapy {1–3 months [61 days (IQR, 46–75 days)], ~ 6 months [180 days (IQR, 170–190 days)], and ~ 12 months [350 days (IQR, 341–360 days)]}, respectively (Fig. 1). ECV and LVEF were measured before (ECV_0_, LVEF_0_), during ((ECV_1_, LVEF_1_) and (ECV_2_, LVEF_2_)), and after (ECV_3_, LVEF_3_) anthracycline treatment, respectively. The CTRCD (+) group represents patients who exhibited CTRCD during chemotherapy for BC and the CTRCD (−) group represents the patients with no CTRCD. Age at first CT examination for all patients was 51.31 ± 9.10 years. Mean hematocrit before chemotherapy was 39.26% ± 3.14%, whereas electrocardiogram or echocardiography showed no abnormal findings.

**Table 1:** Baseline characteristics of the study population

Variable	Entire cohort	CTRCD (−)	CTRCD (+)	value
	(*N* = 1151)	(*N* = 1049)	(*N* = 102)	
Age at cancer diagnosis, years old	51.31 ± 9.10	51.17 ± 8.96	52.75 ± 10.34	0.139
Height, cm	162.46 ± 4.58	162.48 ± 4.53	162.27 ± 5.00	0.663
Weight, kg	57.72 ± 7.49	57.67 ± 7.46	58.26 ± 7.86	0.442
Heart rate, beats/min	68.03 ± 6.93	68.07 ± 6.96	67.54 ± 6.65	0.457
Systolic pressure, mmHg	119.08 ± 10.64	118.99 ± 10.73	119.92 ± 9.71	0.403
Diastolic pressure, mmHg	75.90 ± 7.63	75.89 ± 7.64	76.02 ± 7.48	0.868
*Dyslipidemia, n (%)*	52 (4.52)	47 (4.48)	5 (4.90)	0.845
TG	1.62 ± 1.32	1.63 ± 1.35	1.50 ± 0.95	0.332
TC	4.91 ± 1.04	4.90 ± 1.02	4.94 ± 1.20	0.722
HDL	1.57 ± 0.38	1.58 ± 0.38	1.51 ± 0.35	0.061
LDL	2.73 ± 0.81	2.72 ± 0.80	2.80 ± 0.85	0.365
ALT	25.21 ± 23.44	25.07 ± 23.18	26.67 ± 26.06	0.510
AST	27.97 ± 29.23	27.66 ± 29.23	31.21 ± 29.16	0.241
FBG	4.85 ± 0.64	4.86 ± 0.64	4.83 ± 0.61	0.659
*Histologic type*				0.981
Invasive ductal carcinoma	975 (84.71)	888 (84.65)	87 (85.29)	
Invasive lobular carcinoma	114 (9.90)	104 (9.91)	10 (9.80)	
Intraductal papillary carcinoma	47 (4.08)	45 (4.29)	2 (1.96)	
Mucinous carcinoma	10 (0.87)	7 (0.67)	3 (2.46)	
Other	5 (*p* 0.43)	5 (0.48)	0 (0)	
*Clinical stage*				0.156
I	174 (15.12)	158 (15.06)	16 (15.69)	
II	438 (38.05)	402 (38.32)	36 (35.29)	
III	280 (24.33)	265 (25.26)	15 (14.71)	
IV	259 (22.50)	224 (21.35)	35 (34.31)	
*HER-2*				0.055
Positive, *n* (%)	510 (44.31)	474 (45.19)	36 (35.29)	
Negative, *n* (%)	641 (55.69)	575 (54.81)	66 (64.71)	
*Chemotherapy equivalent dose, mg/m*^*2*^	307.48 ± 11.06	306.73 ± 10.65	307.08 ± 10.08	0.750
Epirubicin, *n* (%)	645 (56.04)	569 (54.24)	76 (74.51)	
Pirarubicin, *n* (%)	506 (43.96)	480 (45.76)	26 (25.49)	
Hematocrit (%)	39.26 ± 3.14	39.27 ± 3.15	39.11 ± 3.03	0.609
LVEF by 2DE, %	65.85 ± 2.72	65.88 ± 2.65	65.57 ± 3.28	0.277

*TG*, triglyceride; *TC*, cholesterol; *HDL*, high-density lipoprotein; *LDL*, low density lipoprotein; *ALT*, alanine transaminase; *AST*, glutamic oxalacetic transaminase; *FBG*, fasting blood-glucose; *LVEF*, left ventricular ejection fraction; *2DE*, 2-dimensional echocardiography

*p*: *p* value between group CTRCD (−) and CTRCD (+)

### Incidence and timing of CTRCD

The mean baseline LVEF of 1151 patients with BC was 65.85% ± 2.72%; no patient had an LVEF less than 55% as measured by 2DE prior to treatment. During chemotherapy and follow-up, a total of 102 (8.86%) patients developed CTRCD. However, during baseline 2DE examination, the mean LVEF of the CTRCD (+) and CTRCD (−) groups were not statistically different (65.88% ± 2.65% vs. 65.57% ± 3.28%, *p* > 0.05). For the 102 patients who developed CTRCD during or after chemotherapy, 3 cases (2.9%) were diagnosed at 3 months, 13 cases (12.7%) at 6 months, and 86 cases (84.3%) at 12 months. When conforming to the CTRCD standard, the mean 2DE LVEF of the CTRCD (+) group was 50.46% ± 3.51%.

### Temporal changes in the myocardial ECV of all patients

**Fig. 3:**
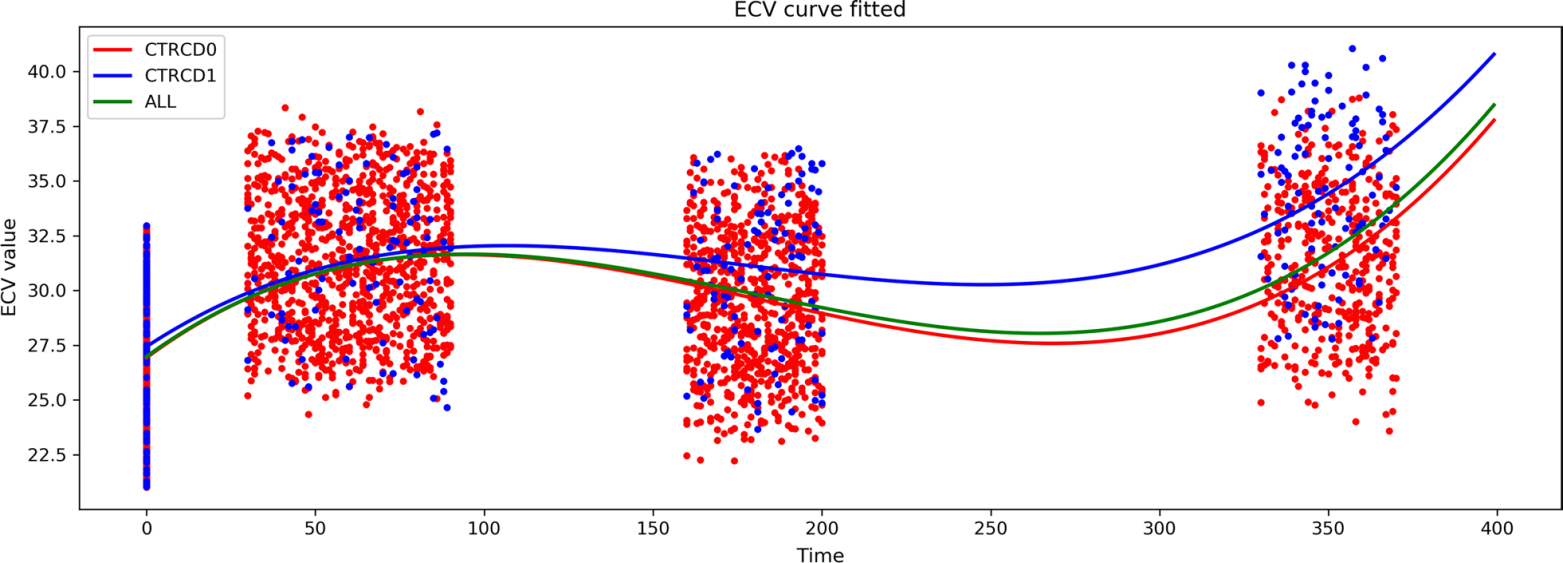
The temporal changes and linear fitting of longitudinal changes of myocardial ECV of BC patients. *CTRCD0*, patients without cancer therapy-related cardiac dysfunction; *CTRCD1*, patients with cancer therapy-related cardiac dysfunction; *ECV*, myocardial extracellular volume; *BC*, breast cancer

Baseline CT examination before anthracycline chemotherapy was performed in 1151 patients; the mean baseline LV myocardial ECV was 26.76% ± 3.03% (ECV_0_: 26.76% ± 3.03%, N_0_ = 1151). At a median interval of 60.5 days (IQR, 45–75 days) from the baseline, a total of 1151 patients underwent the second CT examination during treatment follow-up; the mean ECV_1_ measurement was significantly increased over baseline (ECV_1_: 31.32% ± 3.10%, N_1_ = 1151, *p* < 0.001). A total of 841 patients underwent a third CT scan approximately 180 days (IQR, 171–190 days) from baseline, at which time the patients had completed all cycles of chemotherapy. The mean ECV_2_ (ECV_2_: 29.60% ± 3.24%, N_2_ = 841, *p* < 0.001) was decreased compared to the ECV_1_, but was still a significant increase over baseline (ECV_2_ vs. ECV_0_, *p* < 0.001). After completion of chemotherapy, 511 patients underwent post-chemotherapy CT follow-up, with a median interval time of 350 days (IQR, 341–360 days) from baseline. In addition, it was observed that the value of ECV_3_ was significantly higher than that of ECV_0_, ECV_1_ and ECV_2_ (ECV_3_: 32.05% ± 3.58%, N_3_ = 511, *p* < 0.001). The temporal changes and linear fitting of longitudinal changes in myocardial ECV in all patients are shown in Table 2 and Fig. 3.

**Table 2:** Hematocrit, extracellular volume (ECV) and left ventricular ejection fraction (LVEF) values at different time points

	Baseline	1–3 months	~ 6 months	~ 12 months	*p*^a^	*p*^b^	*p*^c^
	(N_0_ = 1151)	(N_1_ = 1151)	(N_2_ = 841)	(N_3_ = 511)			
Hematocrit (%)	39.26 ± 3.14	38.98 ± 2.16	37.66 ± 3.05	36.57 ± 2.51	0.099	< 0.001	< 0.001
ECV (%)	26.76 ± 3.03	31.32 ± 3.10	29.60 ± 3.24	32.05 ± 3.58	< 0.001	< 0.001	< 0.001
LVEF (%)	65.85 ± 2.72	65.78 ± 2.87	65.55 ± 2.90	63.10 ± 6.51	0.637	0.394	< 0.001

*p*^a^: *p* value of hematocrit, ECV, LVEF compared with the baseline at 1–3 months

*p*^b^: *p* value of hematocrit, ECV, LVEF compared with the baseline at 6 months

*p*^b^: *p* value of hematocrit, ECV, LVEF compared with the baseline at 12 months

### Temporal changes in myocardial ECV of patients with and without CTRCD

**Fig. 4:**
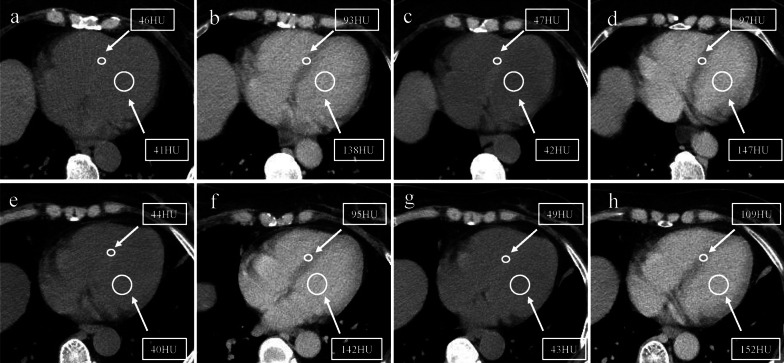
A 48-year-old BC patient without CTRCD (**a**, **b**, **c**, **d**) and a 52-year-old BC patient with CTRCD (**e**, **f**, **g**, **h**). The baseline ECV value before anthracycline chemotherapy in patient without CTRCD was 29.56% derived from unenhanced (**a**) and enhanced CT images (**b**). The ECV value after completing anthracycline chemotherapy was 30.00% derived from unenhanced (**c**) and enhanced CT images (**d**). The baseline ECV value before anthracycline chemotherapy in patient with CTRCD was 29.50% derived from unenhanced (**e**) and enhanced CT images (**f**). The ECV value after completing anthracycline chemotherapy was 34.68% derived from unenhanced (**g**) and enhanced CT images (**h**). *BC*, breast cancer; *CTRCD*, cancer therapy-related cardiac dysfunction; *ECV*, myocardial extracellular volume; *CT*, computed tomography

Across the entire study period, it was found that the temporal changes in LV myocardial ECV were different in patients with and without CTRCD, as shown in Table 3 and Fig. 3. Examples of ECV measurements in two patients with and without CTRCD are shown in Fig. 4. ECV_0_ and ECV_1_ showed no statistical difference between the CTRCD (+) group and the CTRCD (−) group (*p*_1_ = 0.150; *p*_2_ = 0.216). However, ECV_2_ and ECV_3_ showed significant statistical differences between the two groups (*p*_3_ < 0.001; *p*_4_ < 0.001).

**Table 3:** Extracellular volume (ECV) values in group CTRCD (−) and group CTRCD (+) at different time points

	Entire cohort	CTRCD (−)	CTRCD (+)	*p*^1^	*p*^2^	*p*^3^
ECV_0_ (%)	26.76 ± 3.03	26.72 ± 2.99	27.17 ± 3.35			0.150
ECV_1_ (%)	31.32 ± 3.10	31.29 ± 3.05	31.68 ± 3.49	< 0.001	< 0.001	0.216
ECV_2_ (%)	29.60 ± 3.24	29.42 ± 3.17	30.86 ± 3.50	< 0.001	< 0.001	< 0.001
ECV_3_ (%)	32.05 ± 3.58	31.44 ± 3.30	34.51 ± 3.62	< 0.001	< 0.001	< 0.001

*p*^1^: *p* value of ECV compared with the baseline in group CTRCD (−)

*p*^2^: *p* value of ECV compared with the baseline in group CTRCD (+)

*p*^3^: *p* value between group CTRCD (−) and CTRCD (+)

### Reproducibility

A total of 175 patients were assessed for pre-treatment ECVs reproducibility analyses. The CT-derived ECVs yielded excellent intra- and interobserver agreement, with ICC ranging from 0.772 to 0.946.

## Discussion

In a cohort of patients with BC receiving therapy with anthracyclines, the results demonstrated the feasibility of CT-derived ECV as a biomarker of monitoring the early cardiotoxicity of anthracyclines. Notably, in this study, the ECV changed significantly earlier than the observed changes in LVEF. Accordingly, the ECV changes were more sensitive and had a higher diagnostic value than LVEF in monitoring anthracycline-induced cardiotoxicity.

Anthracycline is a representative chemotherapeutic agent and is highly effective against a broad spectrum of malignancies including breast cancer [22]. Given its status as the most commonly used chemotherapy treatment in patients with cancer, the clinical implications of anthracycline-induced cardiotoxicity are expanding [23]. Anthracycline cardiotoxicity can be classified as acute or chronic, with acute toxicity occurring within 2–3 days of administration, whereas chronic toxicity can be detected in early and late stages [24]. Early effects appear within the first year of treatment, while late effects can occur years later [17].

Currently, LVEF or global longitudinal strain is clinically used for early detection of cardiotoxicity [17]; however, these changes reflect severe impairment of myocardial function and, therefore, only manifest themselves at later stages of disease. In recent years, CMR T1 mapping and ECV fraction measurement have been a reliable and noninvasive method for assessing diffuse myocardial fibrosis caused by anthracycline-induced cardiotoxicity [8, 9]. However, CMR imaging is not widely used in clinical practice at all medical institutions. It has been reported that CT-derived ECV was closely related to CMR-derived ECV and histology [14]. We suggested that this is because both iodinated contrast agents for CT and gadolinium contrast agents for MRI are extracellular interstitial agents. Therefore, CT is an attractive alternative to CMR, particularly in those individuals with contraindications to CMR [25]. In addition, our baseline myocardial ECVs are consistent with those reported by Monti et al. [19] and Kurita et al. [26], indicating that the ECVs obtained by chest contrast-enhanced CT is accurate.

Anthracycline-induced cardiotoxicity is believed to be a continuous process, beginning with subclinical myocardial cell damage and leading to early asymptomatic decline in LVEF that can later progress to symptomatic heart failure [27]. Our data on patients with BC are relatively comprehensive; changes in left ventricular myocardial ECVs can be observed throughout the cycle before, during, and after completion of chemotherapy. In our study, we observed a significant increase in ECVs as early as during chemotherapy; specifically, in patients without CTRCD, the myocardial ECV was significantly elevated without detectable changes in LVEF. Furthermore, the ECV changed significantly 3 months after beginning treatment, whereas significant changes in the LVEF were not observed until after 6 months of treatment. Our results were similar to those of a previous animal study in which the mean myocardial ECV increased significantly at week 3, prior to the decrease in LVEF (week 6) [28].

Notably, in our study, we observed a decrease in ECVs at the second follow-up exam (ECV_2_) compared with the first follow-up (ECV_1_). An early anthracycline toxicity animal model reported that degenerative myocardial changes, vacuolation, and interstitial edema, in addition to fibrosis, contribute to the increase in early ECV [29, 30]. According to Ewe et al. [11], anthracycline cardiotoxicity is due to acute edema and subsequent fibrosis, both of which determine an increase in ECV. Namely, myocardial injury preceded the development of fibrosis. Therefore, the lower ECV_2_ compared with ECV_1_ in our study may be caused by the regression of interstitial edema. In other words, ECV might reflect subclinical myocardial injury induced by anthracycline cardiotoxicity prior to myocardial fibrosis and reduced LVEF. However, further studies are required.

In addition, we hypothesized that the mechanism by which anthracycline-induced cardiotoxicity results in changes in myocardial ECV was the induction of myocardial edema during the early stages of treatment [7] and myocardial fibrosis during later stages [30]. In our study, the ECV (ECV_2_, ECV_3_) of the CTRCD group exhibited greater changes than in patients without CTRCD. The median time points at which this change was observed were 180 and 350 days posttreatment, which corresponds to the later stages. These results indicate the myocardial fibrosis caused by anthracycline-induced cardiotoxicity in patients with CTRCD was more serious than that in patients without CTRCD in late stages of treatment. However, there was no significant difference in ECVs (ECV_0_, ECV_1_) between the two groups during the early stage. This might be because the LVEF in the CTRCD (+) group did not decrease significantly in the early stages of chemotherapy, so the change in ECV in the two groups was similar at this time.

Some limitations of this study must be considered. First, this was a monocentric study despite its large sample size. Some patients were excluded due to lack of hematocrit, or respiratory artifacts that greatly affected the measurement of ECVs or they did not undergo baseline examination at our hospital. Second, this was a longitudinal observation experiment, in which ECVs of the left ventricular septum were measured at multiple time points; however, the observation time would have been more informative if it had been longer. Third, given the retrospective nature of the study, a subset of patients was lost to follow-up at each follow-up time point. Finally, it is important to note that the ECVs in our study were measured using a chest contrast-enhanced CT that included images of the heart, not the specific cardiac CT. However, some previous studies have confirmed the feasibility of body enhanced CT in measuring left ventricular ECV [19, 31]. As this was a retrospective study, cardiac biopsy was not performed in patients with CTRCD although it could be performed in the future prospective studies. Despite these limitations, the myocardial ECVs measured in our study were consistent with those reported previously [26], indicating that the ECV obtained by chest contrast-enhanced CT is feasible.

## Conclusions

CT-derived ECV is feasible as a biomarker for monitoring anthracycline cardiotoxicity in patients with BC. In addition, changes in ECV might facilitate the detection of patients experiencing anthracycline cardiotoxicity before LV functional impairment and LVEF decrease becomes evident, thus allowing therapeutic adjustments and consideration of other treatment regimens.

## Data Availability

The datasets generated during and/or analyzed during the current study are available from the corresponding author on reasonable request.

## Abbreviations

2DE: Two-dimensional echocardiography
AC: Anthracycline
BC: Breast cancer
CMR: Cardiac magnetic resonance
CT: Computed tomography
CTRCD: Cancer therapy-related cardiac dysfunction
ECV: Myocardial extracellular volume
HCT: Hematocrit
HU: Hounsfield units
ICC: Interclass correlation coefficient
IQR: Interquartile range
LVEF: Left ventricular ejection fraction
ROI: Region of interest
